# Improved Energetic-Behaviors of Spontaneously Surface-Mediated Al Particles

**DOI:** 10.1038/s41598-017-04758-7

**Published:** 2017-07-05

**Authors:** Dong Won Kim, Kyung Tae Kim, Tae Sik Min, Kyung Ju Kim, Soo Hyung Kim

**Affiliations:** 1 Powder Technology Department, Korea Institute of Materials Science, 797 Changwondaero, Seongsan-gu, Changwon, Gyeongnam 51508 Republic of Korea; 2 Department of Nano Fusion Technology and Nano Energy Engineering, College of Nanoscience and Nanotechnolgy, Pusan National University, 30 Jangjeon-dong, Geumjung-gu, Busan 609-735 Republic of Korea

## Abstract

Surface-mediated Al particles are synthesized by incorporating the stable fluoride reaction of Al-F on a pure Al surface in place of natural oxides. Al particles with fluoro-polymer directly adsorbed on the surface show a considerable capability to overcome limitations caused by the surface oxide. Here, we report that Al fluoride when spontaneously formed at the poly(vinylidene fluoride)/Al interface serves as an oxidation-protecting layer while also providing an efficient combustion path along which the internal Al rapidly reacts with external oxygen atoms. Both thermal oxidation and explosion tests of the poly(vinylidene fluoride)/Al particles show superior exothermic enthalpy energy and simultaneously rapid oxidation reactivity compared to those of Al_2_O_3_ passivated Al particles. It is clearly elucidated that the enhanced energetic properties of Al particles mediated by poly(vinylidene fluoride) originate from the extraordinary pyrolytic process of Al fluoride occurring at a low temperature compared to Al_2_O_3_ passivated Al. Hence, these results clarify that the surface mediation of Al particles can be significantly considered as advanced technology for many energetic applications.

## Introduction

Aluminum (Al) has been considered as a promising reactive metal for energetic applications such as solid rocket fuel, propellants and gas generators given its potentially superior exothermic energy (31.05 kJg^−1^) during oxidation reactions compared to those of other metals^1–5^. However, its use in energetic and electrical fields has often been limited due to the dense oxide layer which forms on its surface. Al spontaneously undergoes a surface passivation process in air, and the oxide, which can reach a thickness of several nanometers^6, 7^, is thermodynamically stable. For example, it is known that Al materials can be easily oxidized even under an extremely low oxygen partial pressure of <10^−50^ atm at 873 K^8^. The surface oxide tends to degrade the electrical conductivity though it also decreases the combustion efficiency. In contrast, if the surface oxide layer is fully removed, it can be expected that a sudden explosion caused by rapid oxidation can occur despite the fact that the physical properties of the Al particles are significantly enhanced^7, 9^. Due to this characteristic of Al, many studies have attempted to develop coating technologies for the surfaces of Al particles to ensure good handling stability and simultaneously to improve its reactivity^10–15^.

As Chung *et al*. and Crouse *et al*. reported, hydrocarbon-based polymers have been investigated for passivating Al particles owing to the feasibility of the coating process as well as the achievement of suitable reactivity^13, 16^. Although hydrocarbon coatings such as epoxides prevent Al particles from surface oxidation at low temperatures, it is well known that their thermal stability is lower compared to when fluoro-polymers are used. Therefore, fluorine-based polymer resins have attracted considerable amounts of attention as passivating materials due to their excellent chemical and thermal stability levels compared to those of other organic materials based on hydrocarbon^17–20^. Fluoro-polymers such as polytetrafluoroethylene (PTFE) and perfluoropolyether (PFPE) have even been shown to be capable of improving the oxidation efficiency of Al particles because fluorine atoms decompose the alumina shell surrounding the Al particles into AlF_3_ products^21–24^. Once the internal Al material is continuously exposed as fluorine-compounds are formed and evaporated, the oxidation reaction proceeds efficiently compared to that with pure Al particles with a dense surface oxide that inhibits continuous combustion^25, 26^.

In this way, polymer materials composed of a carbon backbone with hydrogen and fluorine atoms has been studied as advanced passivation materials for pure Al particles. Simultaneously considering both the reactivity and stability, poly(vinylidene fluoride) (PVDF) which includes the repeating unit (-(CH_2_-CF_2_)_n_-) is expected to offer the advantages of stable coating and high oxidation efficiency by both fluorine and hydrogen atoms, respectively^27^. Because PVDF feasibly swells in an organic solvent, it can be effectively utilized as a molecular-coating agent in the solution compared to the nanoparticle-based polymers such as PTFE suspension^20, 28^. The approximate melting point of PVDF as low as 438 K can also be considered as an advantage of practical applications in the energy or electrodes industries.

Nonetheless, the use of PVDF on the micro-sized Al particles has rarely been reported thus far, except in the case of Al nanoparticles^29^. This is due to the lack of a direct coating technology which prevent surface oxidation on micro-sized Al particles. Similarly, although the coating of PTFE has been reported on Al particles 45 μm in size^24^. There have been no results from research on coatings for fine Al particles with a size ranging from 1 to 10 μm. Thus, we attempted to synthesize advanced Al particles with improved energetic properties while also maintaining the stability of the 5 μm Al particles by developing a feasible coating technology.

Here, we first present a simultaneous synthetic process that removes the surface oxide layer from fine Al particles while also coating the particles with PVDF. The structure of the PVDF layer introduced instead of the surface oxide is analyzed by focusing on the PVDF/Al interface, and the oxidation-mechanism compared to that of Al_2_O_3_ passivated Al particles is suggested.

## Results and Discussion

**Figure 1:**
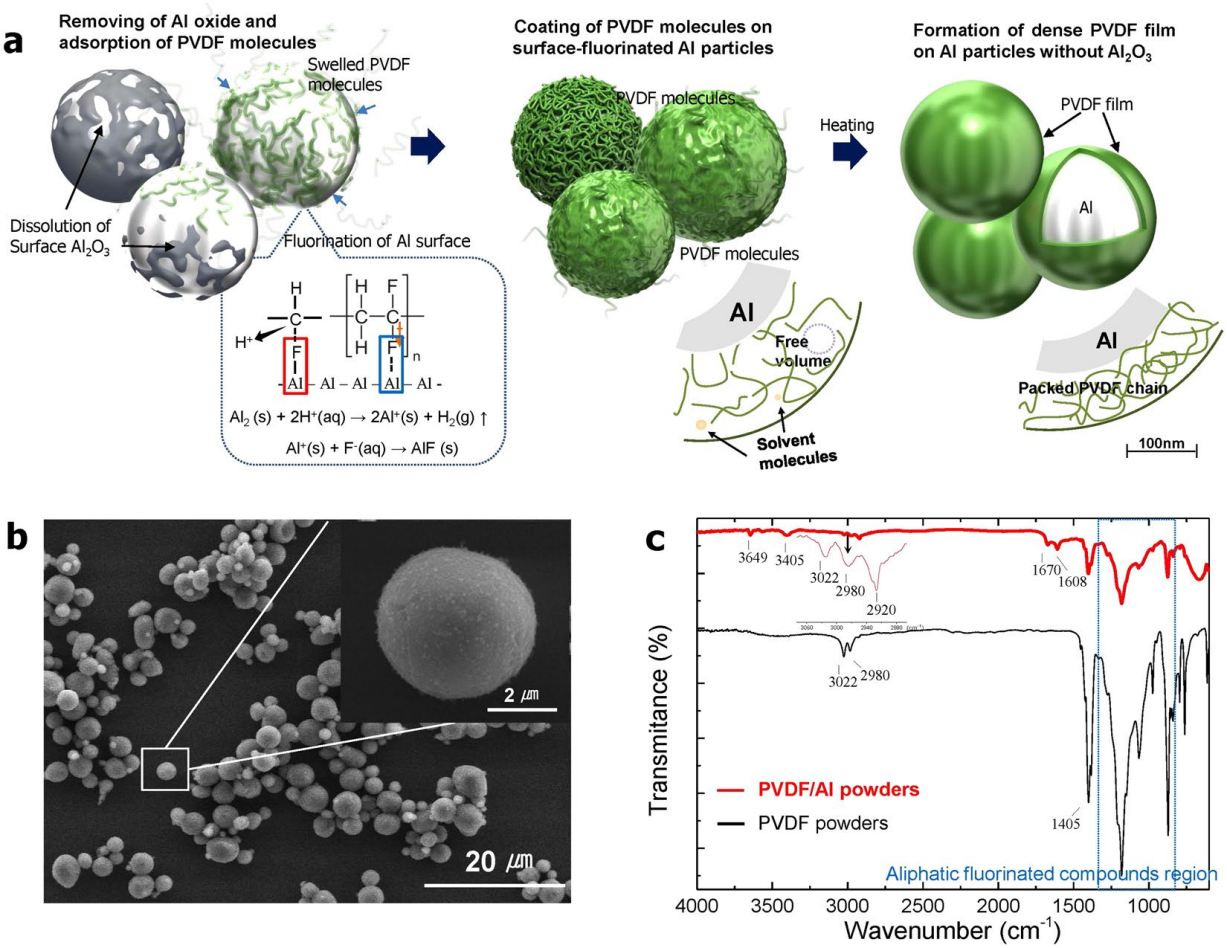
(**a**) Conceptual illustration showing the synthetic process of the PVDF-coated Al particles created via a fluoride reaction on the surface, (**b**) SEM images of the synthesized PVDF/Al powder, and (**c**) transmission FT-IR spectra of the PVDF/Al powder compared to the PVDF.

Figure 1a schematically illustrates the synthetic process for surface-mediated Al particles through a fluoride-based reaction. First, the Al surface oxide is dissolved, becoming AlF_3_ and H_2_O with the use of hydrofluoric acid. The Al surface with the oxide removed reacts spontaneously with fluorine originating from swelled PVDF molecules and HF; that is, the high electron affinity between Al and F atoms allows the PVDF molecules easily to become adsorbed onto the revealed Al surface^30^. At the same time, it is expected that Al-F bonds from F^−^ ion strongly interact with C in the proton-donated PVDF (-CH-CF_2_-) molecules. Thermodynamically, unstable Al tends to exist in the form of AlF_3_ molecules. However, because AlF_3_ is very soluble in an aqueous solution, at about 0.96 g/100 ml at 327 K, it is expected that the AlF_3_ formed on the surface become hydrated and then diffused into the solution. It is designed such that the F atom in the red box and the F atom in the blue box originate from the HF solution and the PVDF, respectively. Thus, in Fig. 1a, the surface with the formation of AlF is mainly illustrated as the partial fluorination of Al. The formed PVDF layer is not fully dense due to the free volume caused by the remaining solvent. Thus, as the final step, a heat treatment is conducted to create a dense layer. From this process carried out in one reactor, PVDF-coated Al (PVDF/Al) particles are synthesized (see Figure S1 in the Supplementary Information).

Figure 1b shows the surface morphology of the synthesized PVDF/Al particles as characterized by FE-SEM. It was confirmed that the coated Al particles have a fine size of approximately 5 μm. As shown in Fig. 1c, the comparison of the FT-IR spectrum between the PVDF/Al particles and the PVDF materials clearly exhibits that the characteristic peaks of the PVDF/Al particles shown in the range of 800 to 1300 cm^−1^ match the aliphatic fluorinated compound area well, while the peaks at 3022 cm^−1^ and 2980 cm^−1^ indicate -CF_2_H functional group stretching and -CH_2_- stretching vibration, respectively. The peak at 2920 cm^−1^ in the PVDF/Al powders represents the -CH_2_Al group, which provides evidence of bonding between the Al and the hydrocarbon. Other FT-IR peaks in the PVDF/Al powder were revealed at 3649, 3505, 1670, and 1608 cm^−1^, correspondingly signifying Al-OH stretching, hydrogen-bonded OH stretching (intramolecular), the aldehyde of the tertiary amide, and OH deformation vibration originating from the very small amounts of water and DMF used as a solvent. These results indicate that the PVDF molecules were spontaneously adsorbed onto the surfaces of the fine Al particles via surface mediation caused by the complex fluoride reactions.

**Figure 2:**
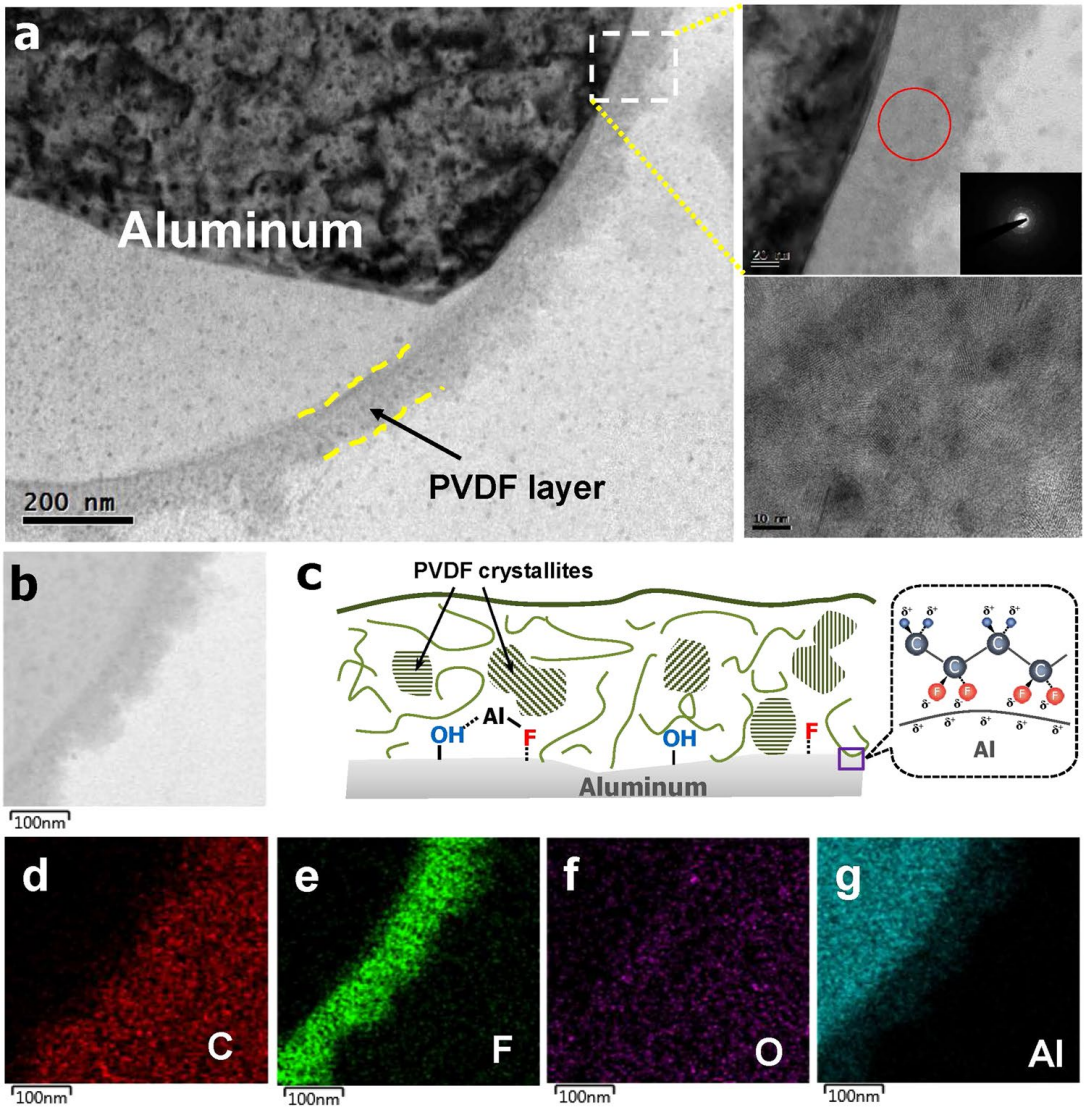
(**a**) Cross-sectional TEM image of a PVDF/Al particle; (**b**) enlarged TEM image of the PVDF-coated region; (**c**) schematic illustration showing the PVDF layer and surface-fluorinated PVDF/Al interface; and EDS results showing the distributions of (**d**) carbon, (**e**) fluorine, (**f**) oxygen, and (**g**) aluminum atoms.

Figure 2a shows a cross-sectional TEM image of the PVDF/Al particles. The coated PVDF shell reveals a uniform layer with a thickness of approximately 100 *nm*. The selected-area electron diffraction (SAED) patterns in the PVDF layer reveal a number of nanocrystals arising from regularly formed PVDF-polymer chains. Unlike the surface Al_2_O_3_ layer with a thickness of a few *nm*, the coated PVDF is approximately 100 *nm* thick on average. It is known that polymer materials essentially tend to be highly permeable to oxygen and moisture due to their chain structure compared to densely packed Al_2_O_3_ film. Creating a long oxygen-diffusion path by controlling the thickness of the polymer coating layer has been considered as an effective means of avoiding re-passivation. Hence, a thicker PVDF coating than the thickness of the surface Al_2_O_3_ is applied to protect the pure Al materials as shown in Fig. 2b. Figure 2c shows a schematic depiction displaying the PVDF structure, which is composed of PVDF crystallites and PVDF molecules.

Figure 2d,e,f and g exhibit the distributions of the carbon, fluorine, oxygen, and Al atoms, respectively, as characterized by EDS equipped in the TEM device. These figures reveal that fluorine atoms are concentrated on the coating layer while oxygen atoms are homogeneously dispersed throughout the particles. Thus, it is likely that PVDF homogeneously surrounds the surfaces of the Al particles.

**Figure 3:**
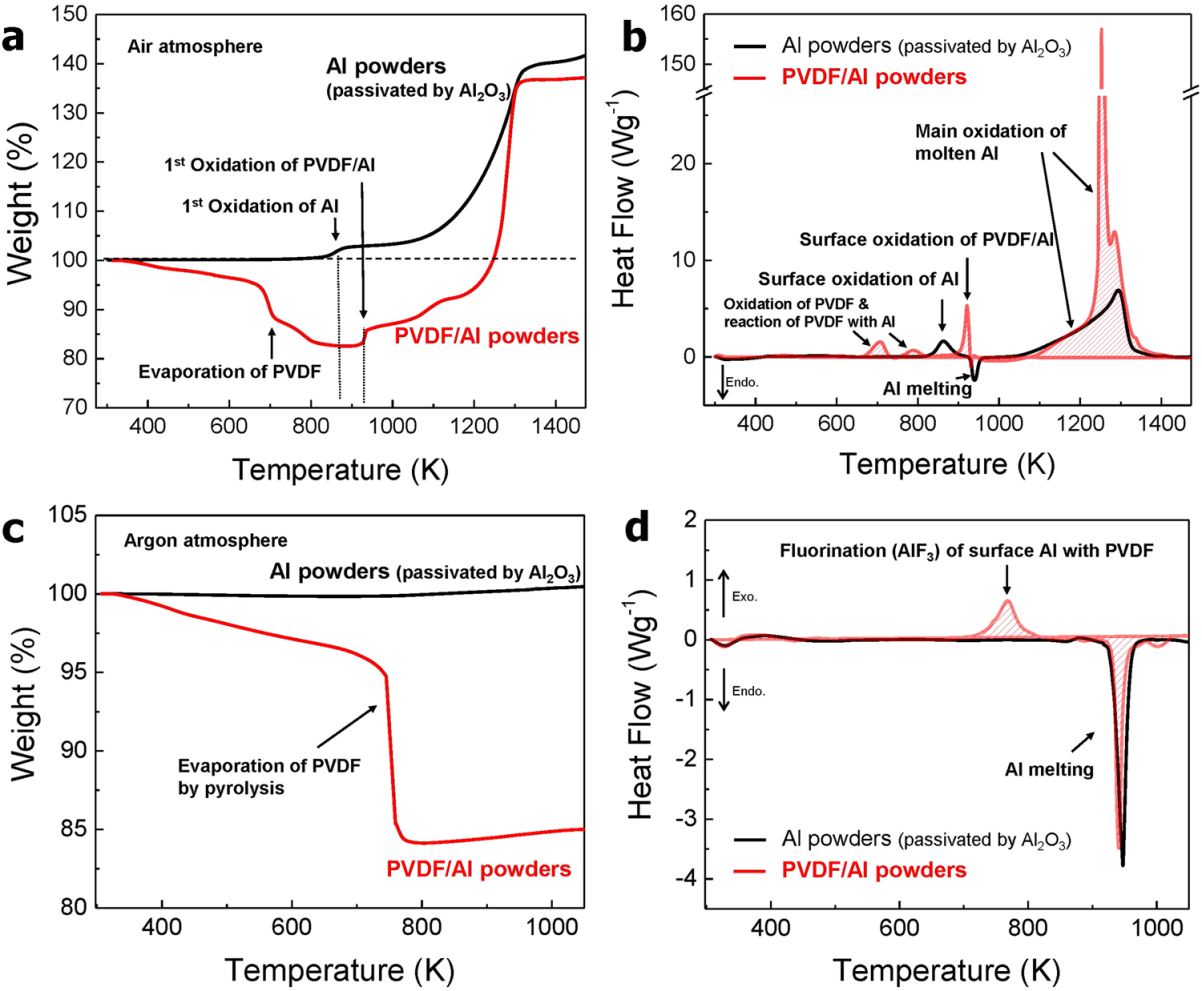
Comparison of the TGA/DSC curves between the PVDF/Al and the Al_2_O_3_ passivated Al powders in (**a**,**b**) air and (**c**,**d**) an argon atmosphere.

Figure 3a shows the results of a thermal analysis in the temperature range of 298 K to 1473 K of the PVDF/Al powder compared to the results with an Al_2_O_3_ passivated Al powder sample. The TGA curves show that the initial weight gain does not lead to any significant difference between the Al_2_O_3_ passivated Al and the PVDF/Al powders up to 373 K, indicating that. The PVDF/Al powder is thermally stable at room temperature. Later, the TGA results showed that the weight of the PVDF/Al powder was decreased by 17.5% up to 823 K compared to that of the Al powder due to the evaporation of the PVDF caused by thermal decomposition and pyrolysis. Weight gain tends to increase in the Al powder and in the PVDF/Al powder at 873 K and 943 K, respectively, which indicates the start of the oxidation reaction on the Al surface. Past a temperature of 1150 K, it was observed that the oxidation of Al leads to rapidly increased weight gain as the surface-coating materials are completely removed in the PVDF/Al powder.

Figure 3b exhibits two small exothermic peaks for the PVDF/Al powder before 823 K stemming from the oxidation of the hydrocarbons during PVDF evaporation step and the formation of AlF_3_ by the surface Al which reacts with PVDF. On the other hand, in the Al_2_O_3_ passivated Al powder, an endothermic peak indicating the melting of Al is observed at 943 K, while in the PVDF/Al powder, this melting is believed to be slightly offset by oxidation occurring at the same temperature. The broad peaks at 1023 K to 1373 K in the Al powder are due to the main oxidation reaction of the molten Al. The sharp peaks of the PVDF/Al powder indicate a more rapid oxidation reaction than the Al powder with a broad peak. Figure 3c and d depict the TGA/DSC curves of the PVDF/Al and Al powder samples in an argon atmosphere. In Fig. 3c, the weight loss of about 4.5% before the pyrolysis of PVDF is due to the evaporation of the pores, solvent and H_2_O of the hydrate in the PVDF layer. Figure 3d clearly shows a small change in the heat flow induced by the fluorination of Al upon the pyrolysis of PVDF at a temperature close to 750 K.

**Figure 4:**
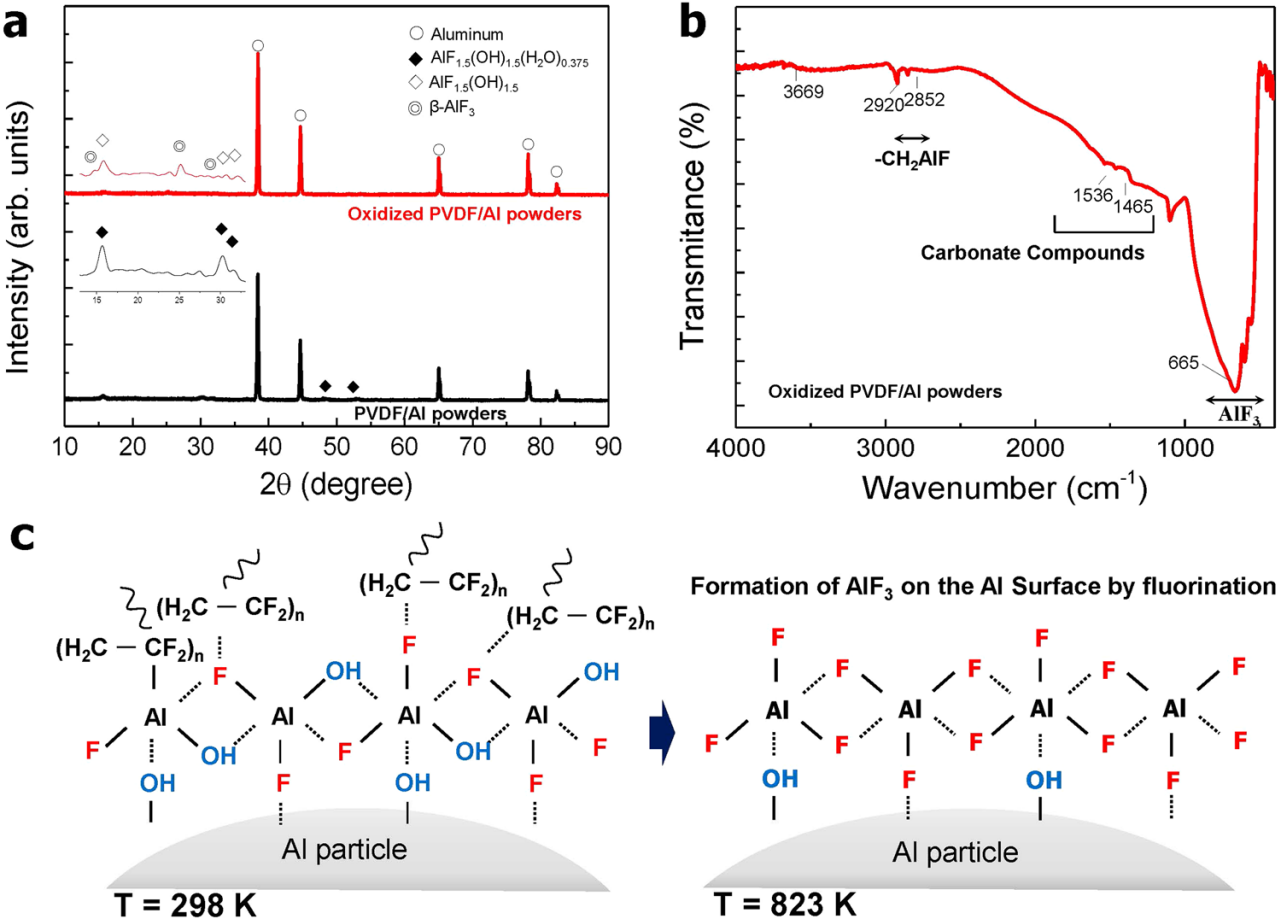
(**a**) X-ray diffraction patterns of the PVDF/Al powder before and after an oxidation treatment at 823 K, (**b**) transmission FT-IR spectrum of oxidized PVDF/Al powder, and (**c**) depictions showing fluorination by thermal oxidation.

Figure 4a shows a comparison of the XRD patterns after the oxidation process. This was done in an effort to understand the characteristics of the chemical compounds produced when PVDF was pyrolyzed on the surface of Al. The XRD patterns shown in Fig. 4a indicate that the representative peaks in the PVDF/Al powder correspond to Al hydroxide fluoride hydrate (AlF_1.5_(OH)_1.5_(H_2_O)_0.375_). On the other hand, the oxidized PVDF/Al particles show peaks at 2θ = 15.8°, 30.7°, and 32.1° indicating the formation of Al hydroxide fluoride (AlF_1.5_(OH)_1.5_), while the additional peaks observed at 2θ = 14.8°, 25.2°, and 29.7° reveal the formation of β-phase Al fluoride (AlF_3_)^31–33^. As reported by DeLisio *et al*., the main pyrolysis product of PVDF is hydrogen fluoride at low heating rates^34^. When the PVDF is removed by increasing the temperature, because the Al-F bond (664 kJmol^−1^) is stronger than the Al-O bond (512 kJmol^−1^), Al fluoride is preferentially formed compared to Al oxide^30^. Figure 4b shows the FT-IR spectrum of the oxidized PVDF/Al powders, where the Al-OH stretching peak can be observed at 3669 cm^−1^ and where -CH_2_AlF group stretching vibrations at 2920 cm^−1^ and 2852 cm^−1^ are confirmed. Furthermore, the transmission peak at 500 to 900 cm^−1^ signifies the Al-F stretching vibration of AlF_3_^32, 35^.

Figure 4c depicts the formation mechanism of Al fluoride, defined as the AlF_3_ phase. Based on the XRD patterns in Fig. 4a, the PVDF layer consisting of some AlF_1.5_(OH)_1.5_∙(H_2_O)_0.375_ at room temperature is thermally decomposed and transformed into the Al fluorides of AlF_1.5_(OH)_1.5_ and AlF_3_ by fluorination. Thus, it is postulated that the reaction between Al and PVDF proceeds as follows^36, 37^:

1 $$2{\rm{Al}}+3{( \mbox{-} {{\rm{CH}}}_{2} \mbox{-} {{\rm{CF}}}_{2} \mbox{-} )}_{{\rm{n}}}\to 3{{\rm{H}}}_{2}+2{{\rm{AlF}}}_{3}+6{\rm{C}}$$

**Figure 5:**
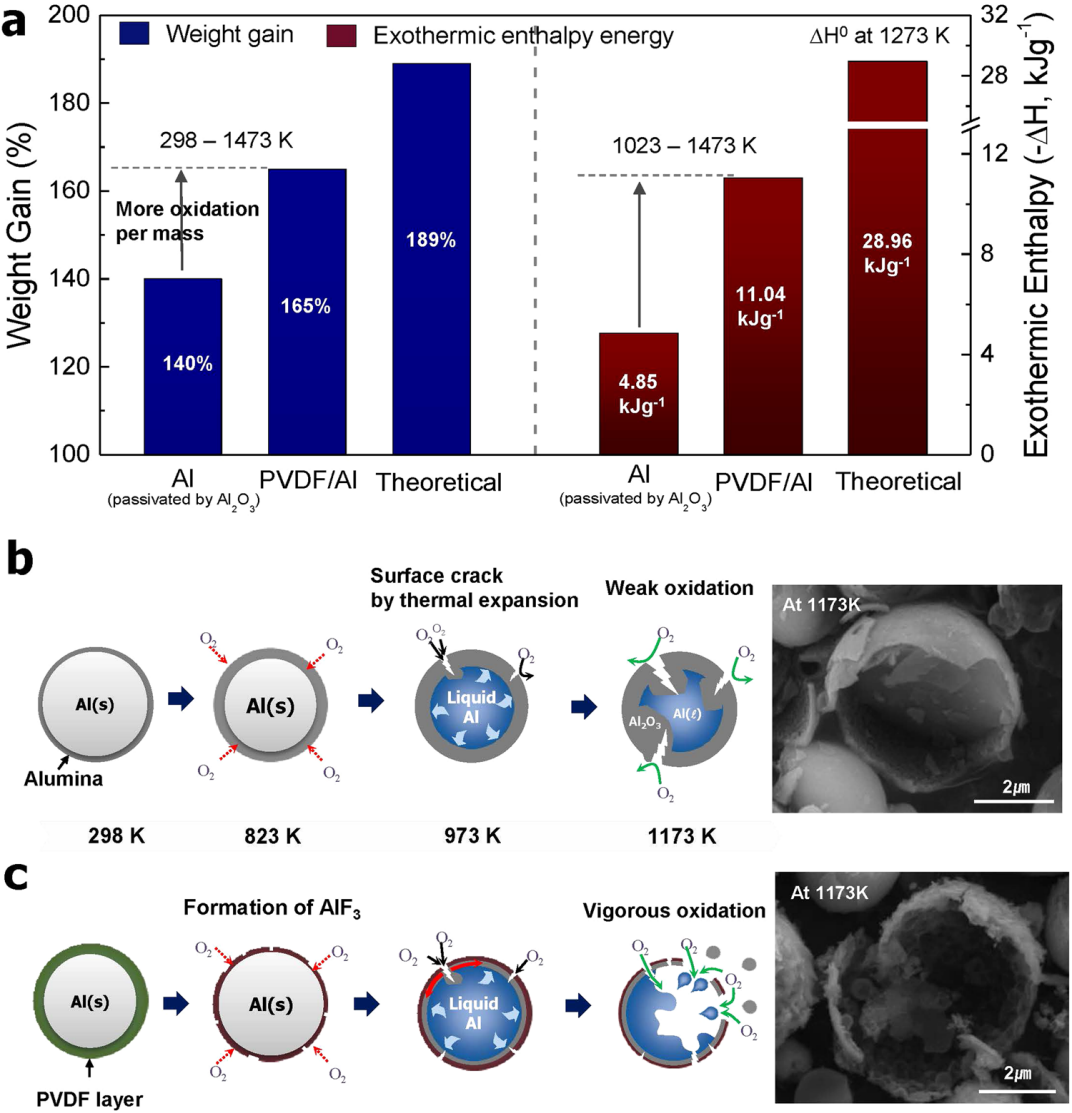
(**a**) Comparison of weight gains and exothermic enthalpy energy levels and a schematic depiction of the proposed oxidation reaction mechanism for (**b**) Al_2_O_3_ passivated Al particles and (**c**) PVDF/Al particles with an increase in the temperature.

Figure 5a shows a comparison of the weight gains and the exothermic enthalpy energy levels of the PVDF/Al powder in the temperature range of 1023 K to 1473 K compared to those of the Al_2_O_3_ passivated Al powder. The weight of the Al powder shows an increase of 140%, and the enthalpy was found to be 4.85 kJg^−1^. Meanwhile, the PVDF/Al powder shows an improved weight gain of 165%, and its exothermic enthalpy energy was determined to be 11.04 kJg^−1^. These results indicate that the PVDF coating provides efficient thermal oxidation per unit of actual Al mass. The enthalpy value in our previous study of PTFE/Al powder was approximately 4.80 kJg^−1^, which is a low value compared to the value of 11.04 kJg^−1^ obtained with the PVDF/Al powder in this study^24^. However, it should be noted that because this result depends fundamentally on the matter of the particle size, the effect of the coating should be compared after synthesizing the PVDF/Al and PTFE/Al particles under similar conditions. The theoretical enthalpy value was determined to be 28.96 kJg^−1^ at 1273 K (see Supplementary Formula 3), and this result is used in a comparison with the experimental results for PVDF/Al and the Al_2_O_3_ passivated Al particles in Fig. 5a.

Figure 5b and c illustrate the overall thermal oxidation behaviors of both powders explained thus far. Figure 5b schematically indicates that the Al_2_O_3_ passivated Al particles experience weak oxidation due to the dense surface oxide layer, and the spontaneously reformed oxide upon an increase in the temperature hinders further oxidation. Thus, a considerable amount of imperfectly oxidized Al remains below 1473 K. Meanwhile, the PVDF/Al particle undergoes oxidation and thus releases vigorous energy, as shown in Fig. 5c. This is due to the formation of feasible diffusion paths which supply external oxygen atoms by the burning or evaporation of Al fluorides formed instead of a dense surface oxide layer (see Supplementary Figures S2 and S3).

**Figure 6:**
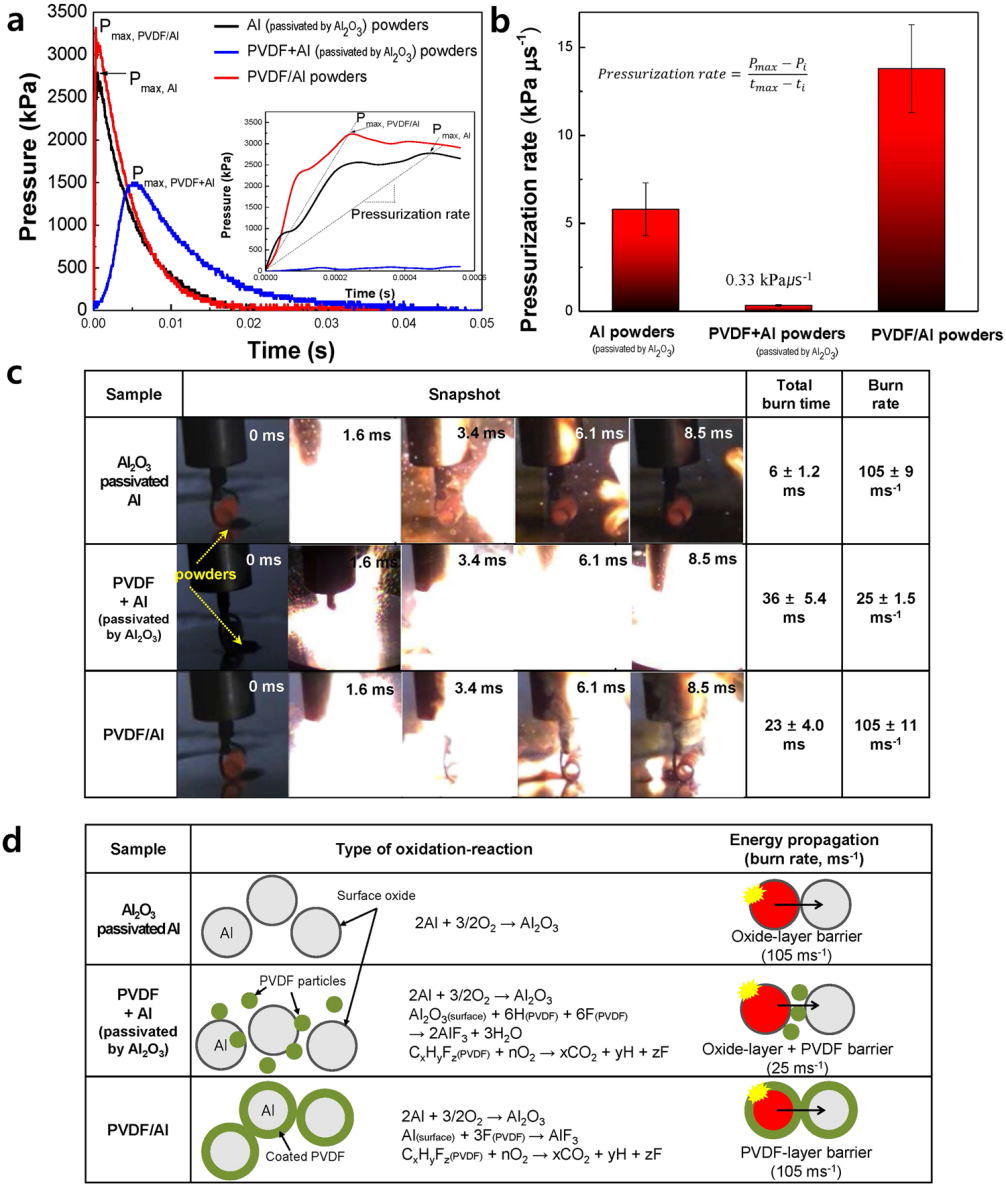
Comparison of (**a**) the pressure change as a function of time, (**b**) the pressurization rate, (**c**) snapshots and measured total burning times obtained from the pressure cell test, and (**d**) the proposed burning mechanism and burn rate as calculated by a high-speed camera of the Al_2_O_3_ passivated Al, the mixed-PVDF/Al and the PVDF/Al powders.

Figure 6a shows the changes in the pressure as a function of time as measured by the flame-ignition of powders in a chamber. Further, a mixture of 17.5 wt% PVDF and 82.5 wt% Al powder was prepared, which is the weight ratio of the PVDF/Al powder. A PCT experiment on the mixing powders was carried out in order to confirm the effect of the PVDF coating. The maximum pressure (*P*_*max*_) of the PVDF/Al powder shows a high value at 3260 kPa compared to the values of 2750 kPa of the Al_2_O_3_ passivated Al and 1500 kPa of the mixed-PVDF/Al powder. The inset graph, enlarged in the time range of 0–60 *μ*s, clearly indicates that the pressure of the ignited PVDF/Al powder reaches *P*_*max*_ within 24 *μs* which is approximately twice as rapid as the time required for the Al_2_O_3_ passivated Al powder. The velocity of the change in the pressure is quantitatively expressed as the pressurization rate (kPa.μs^−1^). This is calculated using equation (2).

2 $${\rm{P}}{\rm{r}}{\rm{e}}{\rm{s}}{\rm{s}}{\rm{u}}{\rm{r}}{\rm{i}}{\rm{z}}{\rm{a}}{\rm{t}}{\rm{i}}{\rm{o}}{\rm{n}}\,{\rm{r}}{\rm{a}}{\rm{t}}{\rm{e}}=({P}_{max}\,{\textstyle \text{-}}\,{P}_{i})/({t}_{max}\,{\textstyle \text{-}}\,{t}_{i})$$

Here, *P*_*max*_ is the maximum pressure, *P*_*i*_ is the pressure when the ignition process begins, *t*_*max*_ is the time until the pressure reaches its maximum value, and *t*_*i*_ is the ignition time.

Figure 6b clearly shows the improvement in the pressurization rate of the PVDF/Al powders. The higher pressurization rate of PVDF/Al (13.8 kPa∙μs^−1^) than that of the Al_2_O_3_ passivated Al (5.8 kPa.μs^−1^) is found to be due to gaseous phases such as CO_2_ and H_2_ generated by the thermal decomposition of PVDF^34^. Interestingly, the pressurization rate of the mixed PVDF/Al powders was very low at 0.33 kPa.μs^−1^. Figure 6c shows a snapshot and the total burning times of the three samples. The burning time of the mixed PVDF/Al powders was approximately 36 ms, which indicated that combustion lasted longer than in the other two powders. Based on this result, Fig. 6d schematically presents the oxidation characteristics of the Al_2_O_3_ passivated Al, the mixed PVDF/Al and the PVDF/Al particles. The burning rate of the Al powder is nearly four times faster than the lowest value of 25 ms^−1^ of the mixed PVDF/Al powder, as displayed in Fig. 6d. That is, the propagation of the energy is affected by the inter-particle distance or by the interfacial materials. Even when one Al particle is ignited in the mixed PVDF/Al powder, the energy propagation can be retarded by surface oxides and PVDF powder acting as barriers to prevent the transfer of heat energy.

For the surface-mediated PVDF/Al particles, there are few surface oxides and only coated-PVDF materials present at the Al surfaces. Therefore, because PVDF directly reacts with the Al rather than the Al_2_O_3_ layer, the reaction is analyzed, with the finding showing that much of the Al is efficiently oxidized. Simultaneously, a long burning time is revealed due to the presence of PVDF materials compared to the Al powder with surface oxides. These properties are modulated in the similar burn rates between the Al_2_O_3_ passivated Al and the surface-mediated PVDF/Al particles. As proof of effective oxidation, the presence of remnant Al particles oxidized at 1173 K was confirmed in a FE-SEM/EDS analysis (see Figures S2 and S3 in the Supplementary Information). From the EDS results, the unreacted Al of the oxidized PVDF/Al powder was calculated and found to account for 6.67 wt%, while the Al_2_O_3_ passivated Al powder amounted to 25.7 wt%.

In summary, the surfaces of fine Al particles were mediated using a fluoride reaction spontaneously induced via a one-pot process under an acidic solution. The synthesized PVDF/Al particles show that the PVDF is directly adsorbed with pure Al due to strong chemical bonds such as Al-F and Al-F-C. It was found for the first time that the main role of Al fluoride is to protect the pure Al surface but also to provide efficient oxidation paths where the inner Al rapidly encounters external oxygen atoms. The results of an energetic analysis confirm that the PVDF/Al particles show superior exothermic energy, a rapid combustion rate and long burning time compared to those of the Al_2_O_3_ passivated Al particles. Thus, it is concluded that these results when utilizing the surface fluorination of Al particles are highly attractive for advanced energetic materials.

## Methods

### Chemicals and Materials

The Al powder used in the experiment has a spherical shape with an average size of 5 μm. The surface oxide is 6.61 *nm* thick (see Supplementary Figure S4). A commercial PVDF powder (average Mw ~534,000 by GPC) purchased from Sigma-Aldrich was used. Hydrofluoric acid (HF, J. T. Baker, 48.0 to 51.0%) was used to remove the alumina shell which surrounded the Al. N,N-dimethylformamide (DMF: C_3_H_7_NO, Sigma-Aldrich, anhydrous, 99.8%) was used as a solvent to dissolve the PVDF.

### Removing the surface oxide and coating of the PVDF

In this step, 30 ml of a mixed aqueous solution was prepared with 15 ml of DMF and 15 ml of 3.0 wt% hydrofluoric acid, after which 1.0 g of Al powder was dispersed into it. Simultaneously, 0.2 g of PVDF powder was put into the DMF. Approximately 5 minutes later, the completely dissolved PVDF solution was obtained and it was poured into an etched-Al suspension, and then the solution of PVDF molecules and etched Al powders was stirred to complete the homogeneous coating reaction. After 4 hours, the synthesized PVDF-coated Al powder was filtered and then washed with methanol and ethanol. The prepared PVDF/Al powder was finally dried in a vacuum oven for 24 hours at 333 K (see the Supplementary Figure S1).

### Analysis and Instruments

A field emission scanning electron microscope (FE-SEM, MIRA II LMH, Tescan) was used to examine the surface morphology of the particles, and the chemical bonding of the PVDF shell coated onto the Al particle was analyzed by Fourier transform infrared spectroscopy-attenuated total reflectance (FTIR-ATR, Thermo Scientific, Nicolet iS5). A thermogravimetric analysis (TGA) and a differential scanning calorimetry analysis (DSC, TA Instruments, Q600) were conducted at a heating rate of 10 Kmin^−1^ from room temperature to 1473 K in air or an argon atmosphere. A dual-beam focused ion beam (DB-FIB, FEI, NOVA200) was used to prepare cross-sectional TEM specimens of a PVDF/Al particle, and the microstructure of the PVDF/Al interface was analyzed with a field emission transmission electron microscope (FE-TEM, JEOL, JEM-2100F) (See Supplementary Figure S5). The phases of the PVDF/Al powders were analyzed with X-ray diffraction (XRD, Rigaku International Co., D/Max-2500VL).

The enthalpy values of the Al_2_O_3_ passivated Al and PVDF/Al particles were calculated from the peak area of the DSC curves. The DSC equipment was calibrated for the temperature and enthalpy using indium as a standard material according to the guidelines in ASTM E967 and ASTM E968. When TGA and DSC were measured, the amount of active Al used for oxidation is controlled at the same weight. That is, if we used 5 mg of Al_2_O_3_ passivated Al powder, an amount of 6 mg of PVDF/Al is used to confirm the effect of PVDF with an identical amount of active Al.

A pressure cell test (PCT) of the PVDF/Al and Al powders was conducted by the flame ignition method in a closed chamber system with an air atmosphere. The flame was generated by the tip of a tungsten wire which was heated by a controlled DC current. The change in the pressure generated by the vigorous oxidation process was measured as a function of time. Furthermore, the total burning time and the burn rates of the powders were monitored at a frame rate of 5 kHz using a high-speed camera (Photron, FASTCAM SA3120 K) installed on the benchtop. The high-speed camera had a maximum frame rate of 1,200,000 fps, a CMOS image sensor of 17.4 mm × 17.4 mm, and a pixel size of 17 μm × 17 μm.

The PCT and burning tests for the PVDF/Al powders were carried out by preparing composite powders containing Al and CuO nanoparticles (NPs) as additive materials for ignition and oxidizer. The average diameters of Al NPs (Nanotechnology, Inc.) and CuO NPs (NT Base, Inc.) are ~80 *nm* and ~100 *nm*, respectively. The mixing ratio of the powders were fixed to Al NPs:PVDF/Al:CuO = 9:21:70 wt%. Also, the Al_2_O_3_ passivated Al particles were prepared by the same method for comparison. All tests were performed more than 5 times and the averaged values with error range were obtained as the results.

### Data availability

The data that support the findings of this study are available from the corresponding author on request.

## Electronic supplementary material

Supplementary information
