# Supplementary material for: Improved Energetic-Behaviors of Spontaneously Surface-Mediated Al Particles
Source: Sci Rep. 2017 Jul 5;7:4659. doi: 10.1038/s41598-017-04758-7 (PMC5498582; doi:10.1038/s41598-017-04758-7)
Supplement: Supplementary file 1 — Supplementary information [file 41598_2017_4758_MOESM1_ESM.doc]

Supplementary Information

Improved Energetic-Behaviors of Spontaneously Surface-Mediated Al Particles

Dong Won Kim^1^, Kyung Tae Kim^1^,*, Tae Sik Min^1^, Kyung Ju Kim^2^, and Soo Hyung Kim^2^

**Contents**

1. Detailed Experimental Procedure
2. Calculation of theoretical enthalpy value for 1273K
3. Comparison of microstructures between the Al_2_O_3_ passivated Al and the PVDF/Al particles after oxidation up to 1173K
4. Characterization of the Al_2_O_3_ passivated Al particle
5. Microstructure of Al and PVDF/Al particles on DB-FIB sample for TEM analysis
6. Reference

**Detailed Experimental Procedure**

Water-soluble DMF is a polar aprotic solvent and it does not have a hydrolysis reaction with hydrofluoric acid which is a weak acid, so it could be used as a stable solvent for the process in this experiment. Also, DMF does not directly induce aluminum oxidation like other hydrocarbon-based solvents, and unlike acids or alcohols, it does not leave protons, which can reduce the loss of ionized aluminum through the difference in reduction potential between aluminum and proton. (Formula 2)

Al_2_O_3_(s) + 3H^+^(aq) + 3F^-^(aq) → Al(OH)_3_(s) + AlF_3_(aq) (1-a)

Al(OH)_3_(s) + 3H^+^(aq) + 3F^-^(aq) → AlF_3_(aq) + 3H_2_O(l) (1-b)

Al(s) + exceed 3H^+^(aq) → Al^3+^(aq) + 3/2H_2_(g) ↑ (2-a)

Al^3+^(aq) + 3F^-^(aq) → AlF_3_(aq) (2-b)

30 ml of mixed-aqueous solution were prepared with 15ml of DMF and 15ml of 3.0 wt% hydrofluoric acid, and then 1.0 g of Al powder was dispersed into it. As it was stirred approximately 5 minutes, the oxide layer was removed. In advance, 0.2 g of PVDF powder was put in 30ml of DMF and dissolved while maintaining a temperature of 323 K. And 5 minutes later, the completely dissolved PVDF solution was obtained and it was poured into the etched-Al suspension, and then the PVDF+Al solution stirred of about 400 rpm for 4 hours at 328 K to performed the homogeneous coating. After the reaction, the PVDF-coated aluminum powder was filtered then washed least five times with methanol and ethanol. The washed PVDF/Al powder was finally dried in the vacuum of 10^-2^ torr for 24 hours at 333 K.


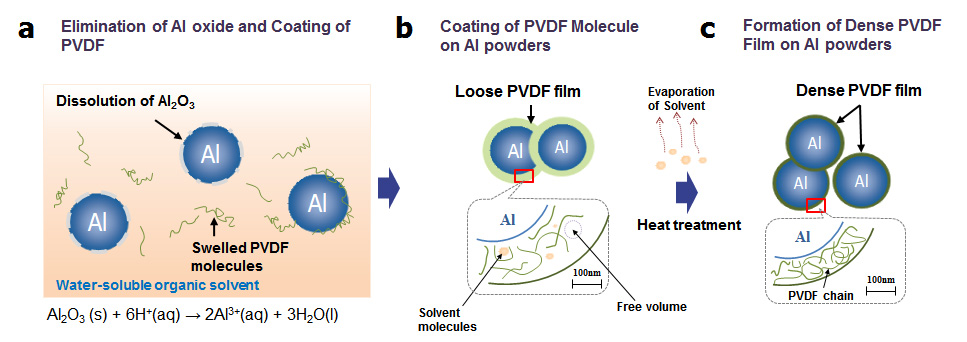


Figure S1. Schematically illustrated one-pot process for PVDF/Al particles.

**Calculation of theoretical enthalpy value for 1273K**

PVDF/Al particle shows meaningful change in heat flow in the temperature range of 1023-1473K. Thus, following equations are used for formation enthalpy value of Al_2_O_3_ at 1273K.


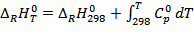
 (3-a)


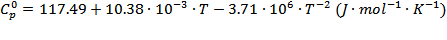
 (3-b)

where the heat of formation (T, 1bar) and standard molar heat capacity (298 K, 1bar) is ‘
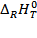
’ and ‘
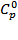
’, respectively. Here,
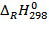
 is a standard heat of formation and the value is -1675.7 ± 1.2 kJmol^-1^. ^[1]^

The calculated enthalpy value is 28.96 kJg^-1^ at 1273 K and the result is displayed to compare with experimental results in PVDF/Al and Al_2_O_3_ passivated Al particle in the manuscript.

**Comparison of microstructures between the Al_2_O_3_ passivated Al and the PVDF/Al particles after oxidation up to 1173K**

The Al_2_O_3_ passivated Al and PVDF/Al powders were oxidized at a slow heating rate of 10 Kmin^-1^ from room temperature to 1173 K in air atmosphere. The oxidized both particles were confirmed by FE-SEM/EDS analysis.

As shown in the FE-SEM image of Figure S2a, the oxidized Al particle can observe the remained aluminum without reacting. The weight of unreacted aluminum calculated was about 25.7wt% from EDS results. However, in case of the PVDF/Al particles, the residual aluminum is hardly observed as shown in Figure S3a. Actually, the unreacted aluminum of oxidized PVDF/Al was about 6.67 wt%.

We analyzed the reason that the Al_2_O_3_ passivated Al particle is not easy to the diffusion of oxygen due to dense surface-oxide layer as commented in the manuscript. Whereas the PVDF/Al particle occur easily the diffusion of oxygen compared to the Al_2_O_3_ passivated Al particle. Hence, it is considered that the PVDF/Al particle will have better combustion efficiency than Al particle at slow heating rates.


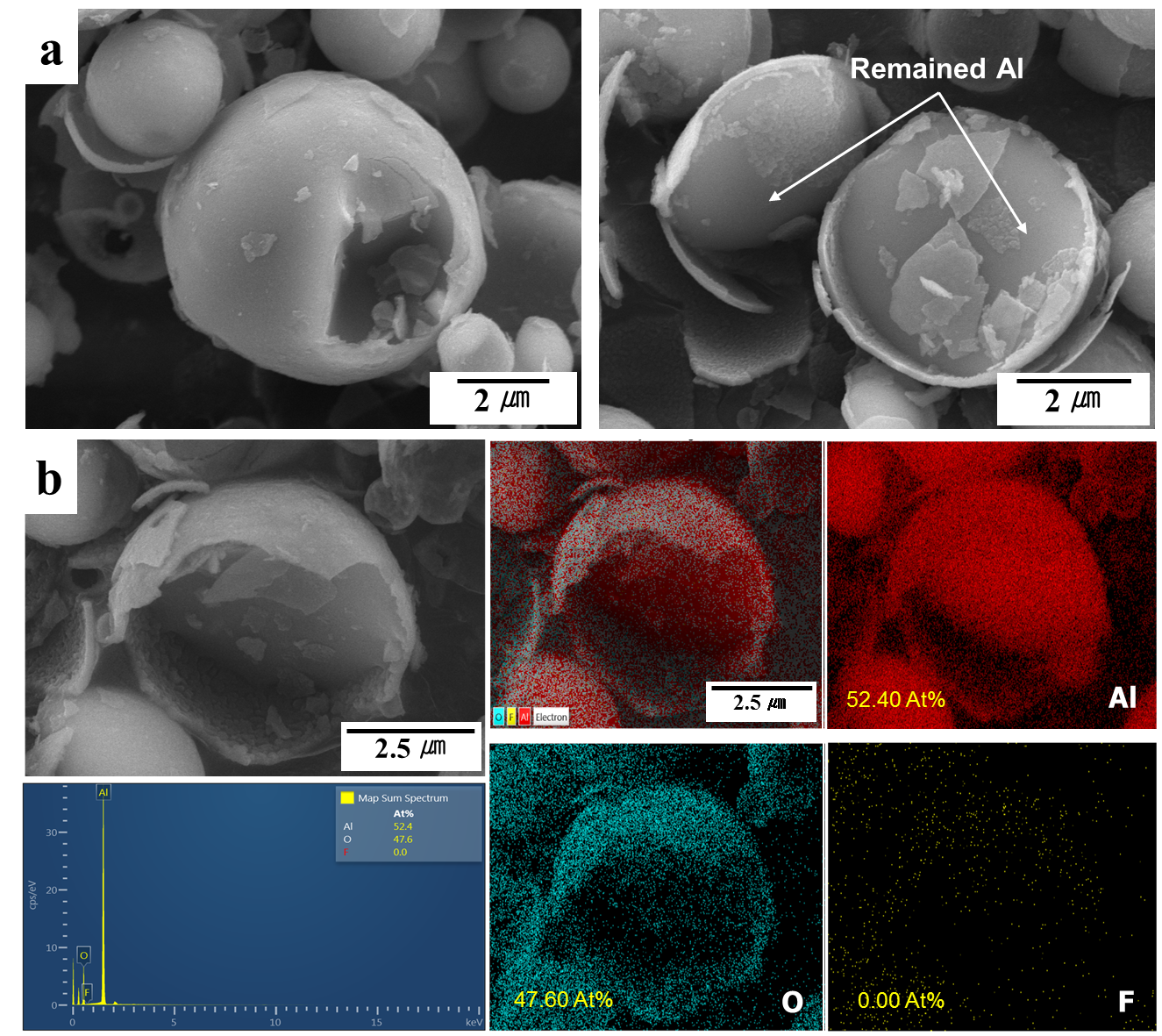


Figure S2. (a) FE-SEM images and (b) EDS results of the Al_2_O_3_ passivated Al particles oxidized from room temperature to 1173 K in air atmosphere.


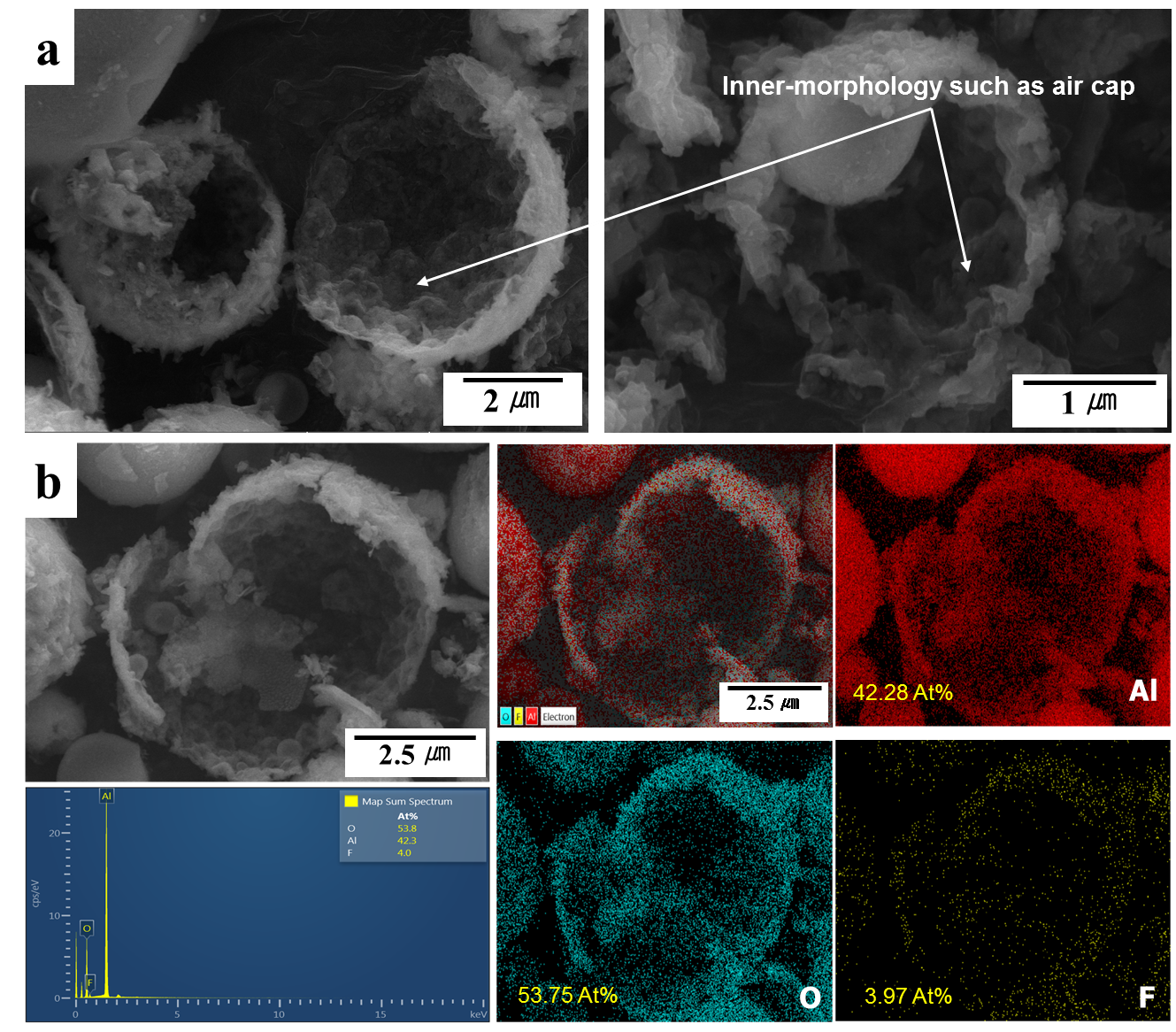


Figure S3. (a) FE-SEM images and (b) EDS results of the PVDF/Al particles oxidized from room temperature to 1173 K in air atmosphere.

**Characterization of the Al_2_O_3_ passivated Al particle utilized in this study**

Figure S4a shows the SEM images of the raw aluminum powder used in this study. The aluminum particles generally exhibit a size distribution of 2 to 7 um. The natural oxide layer thickness of the raw aluminum particle is about 6.6 nm as shown the cross-sectional TEM images of Figure S4b.


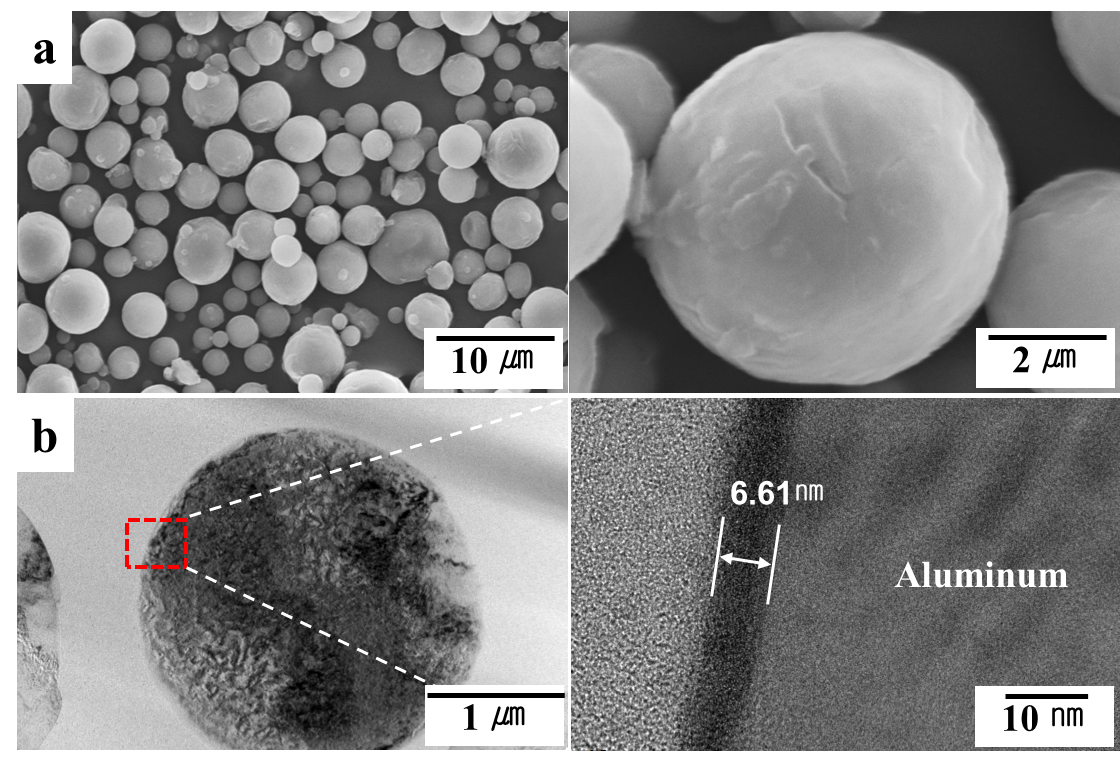


Figure S4. (a) SEM and (b) cross-sectional TEM images of Al_2_O_3_ passivated aluminum particles.

**Microstructure of Al and PVDF/Al particles on DB-FIB sample for TEM analysis**


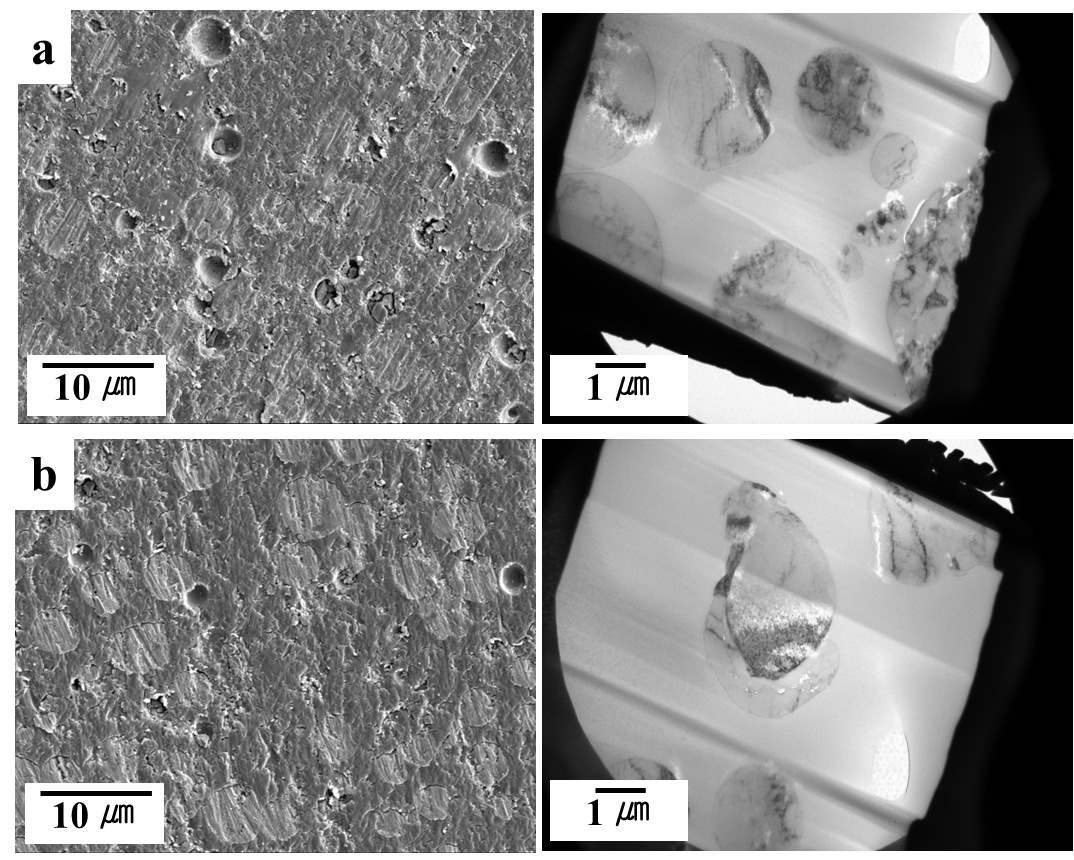


Figure S5. SEM images of cross sectional specimens of (a) the Al_2_O_3_ passivated Al and (b) the PVDF/Al particles prepared by dual-beam focused ion beam (DB-FIB). (Arrows indicate sectioned particles) These samples have been used for checking PVDF layer and interfacial structures.

**Reference**

1. Binnewies, M. & Milke, E. *Thermochemical Data of Elements and Compounds* (Wiley-VCH Verlag GmbH, Weinheim, 2002).
